# Evaluation of different conductive nanostructured particles as filler in smart piezoresistive composites

**DOI:** 10.1186/1556-276X-7-327

**Published:** 2012-06-21

**Authors:** Stefano Stassi, Giancarlo Canavese, Valentina Cauda, Simone L Marasso, Candido Fabrizio Pirri

**Affiliations:** 1 Center for Space Human Robotics, IIT Istituto Italiano di Tecnologia @ PoliTo, C.so Trento 21, 10129, Torino, Italy; 2 Department of Applied Science and Technology, Politecnico di Torino, C.so Duca degli Abruzzi 24, 10129, Torino, Italy

**Keywords:** Spiky nanostructured particles, Shape-controlled synthesis, Quantum tunneling, Piezoresistivity, Polymer-metal composite, Gold, Nickel, Copper

## Abstract

This work presents a comparison between three piezoresistive composite materials based on nanostructured conductive fillers in a polydimethylsiloxane insulating elastomeric matrix for sensing applications. Without any mechanical deformation upon an applied bias, the prepared composites present an insulating electric behavior, while, when subjected to mechanical load, the electric resistance is reduced exponentially. Three different metal fillers were tested: commercial nickel and copper spiky-particles and synthesized highly-pointed gold nanostars. These particles were chosen because of their high electrical conductivity and especially for the presence of nanosized sharp tips on their surface. These features generate an enhancement of the local electric field increasing the tunneling probability between the particles. Different figures of merit concerning the morphology of the fillers were evaluated and correlated with the corresponding functional response of the composite.

## Background

In the last decades, piezoresistive composite materials have found extensive potential application in the fields of micro-sensors [1,2], electromechanical devices, circuit breakers [3], touchable sensitive screens, and tactile sensors for robotics [4], providing cheaper, faster, and more accurate alternatives than the commercially available devices. The properties of these materials could be tuned by varying the nature and the morphology of the particles, used as functional filler and the type of matrix [5]. Several papers report on composites prepared by incorporating different conductive fillers, mostly carbon structures (carbon black and nanotubes) and metal particles in an insulating polymer matrix (e.g., silicones, polyurethane, acrylics, etc.) [6,7]. By varying the type and amount of fillers, the composite can assume the electrical properties of an insulator up to those of a good conductor. In the piezoresistive composites based on tunneling conduction mechanism, a small variation of the external load induces a huge change of the electrical conductivity [8-10]. In these materials, each conductive particle is separated from the others by a thin layer of insulating polymer representing the tunneling barrier [11]. Under the effect of an applied pressure, a mechanical deformation is induced, and the polymer thickness reduces, thus decreasing the tunneling barrier. As a consequence, the probability of tunneling phenomena increases, resulting in a large reduction of the bulk electrical resistance. In these composites, the shape and dimension of the filler particles become as important as the filler nature and amount. In particular, the composites prepared with conductive particles presenting sharp and nanostructured tips on the surface exhibit a huge variation of the electrical conduction in response to a mechanical strain. In fact, this morphology is responsible for a local electric field enhancement [12] that considerably increases the tunneling probability through the insulating barrier.

In this work, we report on the use of three different metal conductive spiky particles into silicone-based polymeric matrix for piezoresistive composites based on tunneling conduction mechanism. These composites were prepared and studied as functional materials for tactile sensors application because of their large sensitivity [13]. The aim of the present work is to understand how the morphological features of the nanostructured particles influence the minimum required amount of the fillers to obtain similar piezoresistive performances among the different composites. In this way, it could be possible to select the best filler and to easily tune the functional properties of the composites in order to reach the required sensor sensitivity.

## Methods

The nickel powder used in this work was supplied by Vale Inco Ltd. (type 123, Toronto, Canada.), copper was obtained from Pometon (LT10, Maerne, Italy) and the bi-component polydimethylsiloxane (PDMS) was purchased by Dow Corning Corporation (SYLGARD 184, Midland, MI, USA). For the synthesis of the gold nanoparticles, all the chemicals were obtained from Sigma Aldrich (St. Louis, MO, USA) and used as received without any further purification.

We have reported in a previous publication [14], the synthetic procedure to prepare shape-controlled, highly pointed, and nanometric-sized gold nanostars. For the composite preparation, the gold nanostars were dispersed in ethanol, and then, the PDMS copolymer was added to the solution [14]. The blend was mixed in an ultrasound bath at 70°C until all the ethanol was evaporated. In the case of nickel and copper filler, the composites were prepared by dispersing the metallic powders in the PDMS by gently mixing, in order to avoid the destruction of the tips on the surface of the particles [9]. The filler to polymer ratio is optimized according to the discussion below. The curing agent was then added to the viscous mixture in a weight ratio of 1:10 with respect to the co-polymer, and the solution was gently mixed at RT. The resulting pastes were outgassed under vacuum for 1 h, poured in PMMA moulds, and then cured in oven at 75°C for 10 h. All the prepared square samples had a footprint of 10 × 10 mm^2^ and the thickness of 1 mm. The resistance of the nickel and copper composite was circa 1 GΩ, in the absence of an applied pressure, while for the gold composite was around 100 GΩ.

**Figure 1:**
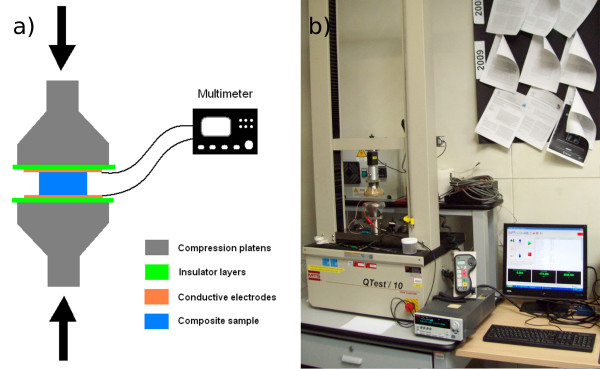
Scheme (a) and photo of experimental setup (b) for electrical resistance measurements under uniaxial compression.

The electro-mechanical characterizations were performed at room temperature with an apparatus composed by a Keithley 2635A sourcemeter (Keithley Instruments Inc., Cleveland, OH, USA) connected to a homemade sample holder and coupled with a universal mechanical testing machine (MTS QTest/10, Eden Prairie, MN, USA) with a load cell of 500 N, as shown in Figure 1. The samples were placed between two Cu plates used as electrodes for applying a voltage in the direction parallel to the applied uniaxial pressure. The voltage was fixed, and the currents were measured coupling them with the applied load. The maximum applied load was 200 N, and the test speed was 0.3 mm/min.

The morphological characterization was carried out by a field emission scanning electron microscope (FESEM, Zeiss SupraTM 40, Oberkochen, Germany). For each metal filler, circa 100 particle tips where measured from the FESEM images in term of radius of curvature (R_tip_) and aspect ratio between the tip height (H_tip_) and its full width at half maximum (FWHM). In order to evaluate the sharpness of the spiky particles, the ratio between the H_tip_ and the core particle diameter (D_core_) was also calculated.

## Results and discussion

**Figure 2:**
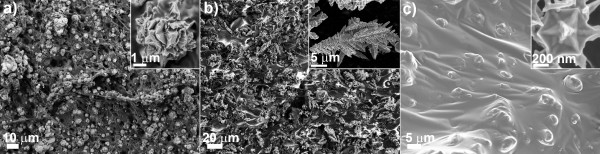
**Scanning electron microscopy images of different PDMS-metal composites and of nanoshaped-spiky particles in the insets.(a) Nickel, (b) copper, and (c) gold.**

**Figure 3:**
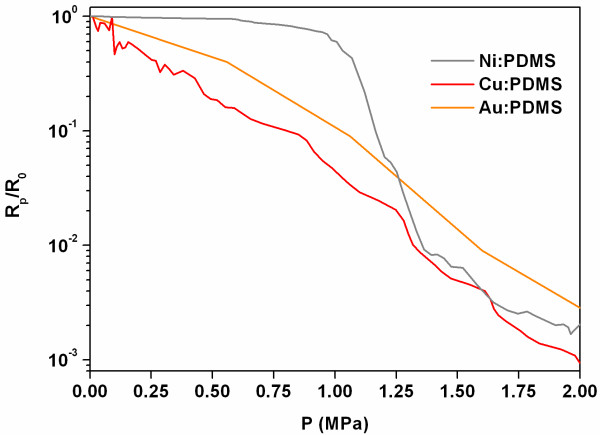
Electric resistance variation of the piezoresistive composites as a function of the applied uniaxial pressure.

In the present paper we, report on the piezoresistive performances of three metal conductive composites, with different fillers, i.e., nickel, copper, and gold (Figure 2). Based on our past experience [9,14], here we compare the results, in terms of electric resistance variation as a function of the applied mechanical pressure, obtained for the optimized compositions of the final composites. In the past works, we tried indeed different weight ratios between the filler amount and the polymeric matrix, ranging from 1:1 to 5.5:1 (filler: PDMS). Here, we are able to identify the minimum weight amount per each kind of filler, required to obtain an appreciable and comparable tunneling conduction effect between the different composites. Therefore, comparable electric resistance variations as a function of the applied mechanical pressure (Figure 3) were obtained with a weight ratio of 3:1 for the Ni: PDMS, 1.75:1 for Cu: PDMS, and 1:1 for the Au: PDMS composites (see also Table 1). In contrast, lower filler to polymer ratios with respect to the previously indicated ones showed an insulating behavior. In order to evaluate the strain sensitivity of the composites, we calculated the gauge factor with the method reported in the work of Abyaneh and Kulkarni [10]. We obtained the values of approximately 18 for the nickel-based composite, and approximately 10 for the copper and gold ones. These gauge factors could be increased by enhancing the content of metallic filler in the polymeric matrix.

**Table 1:** Figures of merit of the nanoshaped-spiky fillers

**Metal particles**	**R**_**tip**_^**a**^**[nm]**	**H**_**tip**_**/FWHM**^**b**^	**H**_**tip**_**/D**_**core**_^**c**^	**Filler: PDMS weight ratio**
Ni	43	1.1	0.09	3.1
Cu	975	3.6	0.37	2:1
Au	17	2.3	0.34	1:1

^a^Average tip radius; ^b^aspect ratio between the H_tip_ and FWHM); ^c^ratio between the H_tip_ and the D_core_.

The three selected particles have different size and shape, ranging from spiky micrometric-sized nickel particles (average diameter, 4.5 μm, Figure 2a), to elongated multi-branched copper ones (average diameter, 12 μm, Figure 2b) up to gold nanosized stars (average diameter, 450 nm, Figure 2c). It was already reported in the literature [8] that highly pronounced and elongated tips at the particle surface increase the electric conductance throughout the composite, amplifying the electric field and, thus, the tunneling probability among the spiky fillers. It is therefore clear that the lower the curvature radius of the conductive tips, the higher the tunneling conductance effect in the composite.

**Figure 4:**
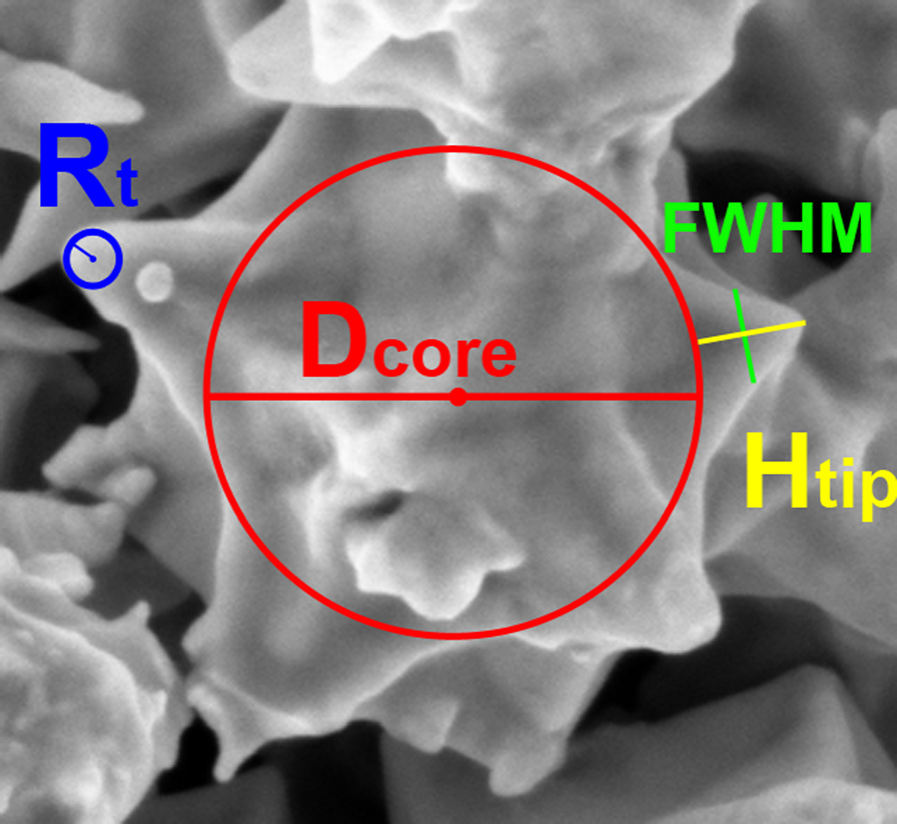
**The scheme of the geometric parameters reported in Table**1.

Based on these FESEM images, we have therefore calculated the different figures of merit describing the morphological features of our spiky particles (see Table 1), as also schematically reported for clarity in Figure 4. Both nickel and gold show nanostructured tips with a small R_tip_ (43 and 17 nm, respectively). However, in the case of nickel, the tips are quite short with respect to the particle micrometric size, thus the H_tip_/D_core_ ratio results very low (0.09). In contrast, very similar H_tip_/D_core_ ratio are obtained for both copper and gold spiky-particles (0.37 and 0.34, respectively).

Considering the obtained values of electric resistance, one can observe a strong relationship between the morphological data calculated here and the used filler amount in the final composite. First, the copper and gold-based composites have both shown remarkable tunneling conduction values at lower filler amount (1.75:1 for Cu: PDMS and 1:1 for Au: PDMS) with respect to the weight ratio used for the nickel-based composite (3:1). We attribute this effect to the higher H_tip_/D_core_ ratio obtained for both copper and gold with respect to the nickel one.

Additionally, another morphological parameter was calculated, that is the aspect ratio between the H_tip_ and its FWHM. Higher is this value, sharper and more slender is the tip. We note that both copper and gold fillers have a higher H_tip_/FWHM ratio than the nickel one.

The combination of both parameters, i.e., high H_tip_/FWHM and H_tip_/D_core_ ratios, implies the presence of sharp tips showing a pronounced height with respect to the core size of the particles. This means that a lower amount of material in weight is required for obtaining similar conductance values, since the probability of the tip to form a tunneling conduction is higher with respect to massive, spherical shaped nanoparticles with the same size.

In addition, the gold-based composite requires an even lower filler amount (1:1) than the copper one (1.75:1) to obtain similar tunneling conductance values. We note, however, that the H_tip_/D_core_ ratio of gold is slightly smaller (0.34) than those of copper (0.37) as well as the H_tip_/FWHM ratios (2.3 for gold versus 3.6 for copper). Thus, the lower radius of curvature of the gold tip shows to play here a predominant role in the tunneling conduction enhancement. Indeed, the R_tip_ of gold is about 60× smaller than that of copper. It is therefore clear that, in the case of gold, the presence of either high H_tip_/D_core_ and H_tip_/FWHM ratios and small R_tip_ is a fundamental prerequisite for obtaining high tunneling conductance value with a low filler to polymer weight ratio. We note in addition that the use of gold has several advantages with respect to the copper and the nickel fillers. First, it was synthesized and *ad-hoc* prepared, whereas both Cu and Ni-particles were obtained commercially. This allows a full control on the size and shape of the gold nanostars with a very good reproducibility, which cannot be reached with the other two metals. Despite higher cost of the starting precursor chemical, its nanometric size and tip nanostructuration allow the use of gold fillers in small amounts in the composite, thus obtaining comparable piezoresistive performances than the other composites with commercial fillers. Furthermore, gold is a safe material (whereas nickel particles were reported to be carcinogenic [15]) and shows higher resistance to oxidation with respect to both nickel and copper. In addition, thanks to the small content of the filler required and its nanometric size, the gold nanostars can be used to prepare very flexible, light, and thin composites, ideal for the integration in MEMS-like technology in tactile sensor applications.

## Conclusions

We have reported on the influence of the filler morphological features on the piezoresistive performances of three different conductive spiky-particle polymeric composites. Based on our previous experience, we have tested different weight ratios of the filler in the PDMS matrix. The aim was to find the minimum amount of nickel, copper, and gold for obtaining comparable tunneling conductance values of the piezoresistive composite as a function of the applied mechanical pressure. We have experimentally observed a strong dependence of the minimum filler amount (for tunneling conduction mechanism of the composite) from the morphological figures of merit. We have found out that particles with sharp tip and small core size, i.e., high H_tip_/D_core_ and H_tip_/FWHM, together with a small curvature radius of the tip (R_tip_), present strong enhancement of the tunneling conduction. Thus, our synthesized gold nanostars showed very good performances in terms of tunneling conductance at a low weight ratio in the composites. Thanks to their nanometer size and nanostructured shape, it is possible to obtain flexible, thin, and light-weight performing piezoresistive composites, which will be well adaptable to tactile sensor application.

## Competing interests

The authors declare that they have no competing interests.

## Authors’ contributions

SS and GC conceived the study and prepared the composites; SS and VC carried out the synthesis of the gold nanostars and drafted the manuscript. GC and SLM performed the measurements and CFP coordinated the study and revised the manuscript. All authors read and approved the final manuscript.
